# Hypertensive Nephrosclerosis: Pathological Changes and Overlap with Diabetic Nephropathy

**DOI:** 10.7759/cureus.92949

**Published:** 2025-09-22

**Authors:** Hussein Qasim, Mahfouz Ktaifan, Ahmad Awawdeh, Karis Khattab, Matteo Luigi Giuseppe Leoni, Giustino Varrassi

**Affiliations:** 1 Department of Pathology and Laboratory Medicine, Jordan University of Science and Technology, Irbid, JOR; 2 Department of Medicine, College of Medicine and Health Sciences, An-Najah National University, Nablus, PSE; 3 Faculty of Medicine, Jordan University of Science and Technology, Irbid, JOR; 4 Department of Medical and Surgical Sciences and Translational Medicine, Sapienza University, Rome, ITA; 5 Department of Pain Medicine, Fondazione Paolo Procacci, Rome, ITA

**Keywords:** chronic kidney disease, diabetic nephropathy, end-stage renal disease, glomerulosclerosis, histopathology, hypertensive nephrosclerosis

## Abstract

Hypertensive nephrosclerosis (HN) and diabetic nephropathy (DN) are the leading global causes of chronic kidney disease (CKD) and end-stage renal disease (ESRD). Both conditions share overlapping clinical and histopathological features, particularly in patients with concurrent hypertension and diabetes, making accurate differentiation challenging. This narrative review synthesizes current evidence on the pathophysiology, histopathology, diagnostic criteria, and therapeutic implications of HN and DN, with emphasis on areas of morphological and clinical overlap. HN is driven by chronic vascular injury from sustained hypertension, resulting in ischemic glomerulosclerosis, arteriolar hyalinosis, and interstitial fibrosis. DN, in contrast, is primarily mediated by hyperglycemia-induced metabolic and hemodynamic stressors, producing diffuse and nodular mesangial expansion, glomerular basement membrane thickening, and bilateral arteriolar hyalinosis. While certain histological features, such as Kimmelstiel-Wilson nodules and efferent arteriole involvement, favor DN, mixed lesions are common in long-standing type 2 diabetes with hypertension. Accurate differentiation is clinically significant, as it guides the application of disease-specific interventions such as intensive glycemic control and emerging pharmacotherapies for DN, versus targeted vascular risk reduction in HN. Future advances in biomarkers, imaging, and genomics hold promise for earlier, noninvasive diagnosis.

## Introduction and background

Diabetic nephropathy (DN) caused by diabetes mellitus is the single most common cause of end-stage renal disease (ESRD) globally [1]. In many regions, hypertensive nephrosclerosis (HN), kidney damage attributable to chronic hypertension, is the second leading cause of ESRD after DN [2]. Given the rising prevalence of type 2 diabetes and hypertension, these conditions frequently coexist in patients, contributing to a large burden of chronic kidney disease (CKD) [3]. Differentiating DN from HN is of significant clinical importance [3]. Each entity has distinct pathogenic mechanisms and characteristic pathological changes, and their management can differ [3]. DN is typically managed with intensive glycemic control and therapies targeting metabolic and hemodynamic pathways of diabetes, whereas HN management centers on aggressive blood pressure control and mitigating vascular injury [4]. Furthermore, prognostic trajectories may vary: kidney disease due to diabetes often progresses more rapidly to ESRD than primary hypertension-related kidney disease, especially if hyperglycemia is not controlled [5]. However, in practice, distinguishing DN from HN can be challenging [6]. Many patients with diabetes also have hypertension, and both conditions can cause overlapping clinical and histological features of kidney damage [7]. Indeed, a substantial subset of diabetic patients may develop renal impairment from non-diabetic causes (such as HN or other glomerulopathies), and conversely long-standing hypertension can cause nephrosclerosis that mimics diabetic changes [8]. The term "hypertensive nephrosclerosis" itself encompasses heterogeneous pathogenic processes and may mask the underlying primary renal diseases (for example, APOL1 gene-associated nephropathy) that coincide with hypertension [9]. The global burden of CKD attributable to HN and DN is substantial and growing. Worldwide, CKD affects approximately 9%-13% of the population, with diabetes and hypertension being the predominant risk factors [10]. In high-income countries, DN accounts for up to 50% of incident ESRD cases, whereas in parts of Africa and the Caribbean, HN is more common, particularly in individuals with APOL1 risk alleles [11]. Low- and middle-income countries are experiencing a rapid rise in both conditions due to increasing prevalence of metabolic syndrome, obesity, and aging populations [12]. Differentiating between HN and DN is not only clinically relevant but also essential for public health planning, as misclassification can skew epidemiologic estimates and resource allocation. Population-based biopsy series suggest that up to one-third of diabetic patients with proteinuria have non-diabetic kidney disease or mixed pathology, which may respond differently to treatment [12]. This review will discuss the pathophysiology and histopathological features of HN and DN in detail, examine the areas of overlap in their pathology, and outline approaches to diagnostically differentiate the two. We also explore the clinical implications of distinguishing HN from DN in terms of treatment decisions and outcomes, and highlight future directions including emerging biomarkers, advanced diagnostic tools, and genetic insights that may improve our ability to accurately identify the predominant etiology of kidney damage in patients with both hypertension and diabetes.

## Review

Methods

This narrative review utilizes a descriptive thematic approach to examine the clinical, pathological, and therapeutic distinctions and overlaps between HN and DN. The review adheres to the Scale for the Assessment of Narrative Review Articles (SANRA) guidelines to ensure quality, transparency, and methodological rigor. Eligible literature included peer-reviewed articles addressing the pathophysiology, histopathology, diagnostic approaches, and treatment strategies for HN and DN, with specific attention to studies analyzing the overlapping lesions or mixed pathology. Acceptable study types included original research, systematic reviews, meta-analyses, case series, and expert commentaries. Only full-text articles published in English were included. Studies were excluded if they lacked relevance to the differential diagnosis of HN and DN, did not provide clear pathological or clinical correlations, or were not peer-reviewed. A comprehensive literature search was conducted using PubMed, Scopus, Embase, Web of Science, and Google Scholar. Search terms were structured with Medical Subject Headings (MeSH) and Boolean operators to enhance precision. The primary search string was: (“hypertensive nephrosclerosis” OR “arterionephrosclerosis” OR “hypertensive renal disease”) AND (“diabetic nephropathy” OR “diabetic kidney disease”) AND (“histopathology” OR “pathophysiology” OR “diagnosis” OR “differential diagnosis”). The reference lists of selected publications were manually screened to identify additional relevant studies not retrieved in the initial database queries.

Pathophysiology of hypertensive nephrosclerosis

Chronic hypertension induces progressive renal injury known as hypertensive nephrosclerosis (HN), primarily through sustained elevation of systemic and intraglomerular pressure [13]. This leads to vascular remodeling and ischemic injury, marked by fibroelastic intimal hyperplasia and hyaline arteriolosclerosis, particularly in afferent arterioles [14]. The resultant luminal narrowing compromises glomerular perfusion, triggering ischemic glomerulosclerosis, tubular atrophy, and interstitial fibrosis [15]. Glomerular hemodynamics are also altered [15]. Initially, autoregulatory vasoconstriction protects glomeruli, but over time, focal loss of autoregulation allows systemic pressures to damage glomerular capillaries [16]. This leads to glomerular hypertrophy and hyperfiltration in surviving nephrons, resulting in focal segmental glomerulosclerosis (FSGS) in addition to ischemic global sclerosis [17]. The activation of renin-angiotensin-aldosterone system (RAAS) further exacerbates injury [18]. Reduced perfusion stimulates renin release, with angiotensin II promoting vasoconstriction, inflammation, and fibrosis via AT₁ receptor-mediated pathways [18]. Aldosterone contributes similarly, while persistent pressure and RAAS signaling promote epithelial injury and epithelial-to-mesenchymal transition (EMT), advancing interstitial fibrosis [19]. Arteriolar narrowing and capillary rarefaction also lead to hypoxia-driven inflammation and fibroblast activation [20]. Other factors, including aging, atherosclerosis, and dyslipidemia, compound renal damage [21]. In malignant hypertension, severe vascular injury (fibrinoid necrosis and “onion-skin” arteriolosclerosis) causes cortical infarcts and acute kidney injury [22]. Genetic susceptibility, particularly APOL1 variants in African ancestry populations, significantly increases the risk and severity of HN [23]. Endothelial dysfunction is increasingly recognized as a central mediator of HN progression. Reduced nitric oxide bioavailability, coupled with increased endothelin-1 production, promotes sustained vasoconstriction, leukocyte adhesion, and smooth muscle proliferation [24]. Oxidative stress further exacerbates injury through the generation of reactive oxygen species (ROS), activating the nuclear factor kappa-light-chain-enhancer of activated B cells (NF-κB) and upregulating monocyte chemoattractant protein-1 (MCP-1), which drives inflammatory cell infiltration [25]. Dyslipidemia and obesity synergize with hypertension to accelerate arteriosclerosis and interstitial fibrosis [26]. In malignant hypertension, these processes are markedly amplified, leading to fibrinoid necrosis and “onion-skin” arteriolopathy within weeks [27].

Histopathological features of hypertensive nephrosclerosis

HN results from chronic systemic and intraglomerular hypertension, leading to progressive vascular and glomerular remodeling [2]. Prolonged high blood pressure induces fibroelastic intimal hyperplasia and arteriolar hyalinosis, particularly in afferent arterioles [28]. These changes narrow the vascular lumen, reducing blood flow to glomeruli and causing chronic ischemia, glomerular collapse, and ultimately glomerulosclerosis [29]. Tubular ischemia similarly contributes to atrophy and interstitial fibrosis [30]. Initially, the kidney attempts to autoregulate glomerular pressure, but focal failure of this mechanism exposes some nephrons to systemic pressures, resulting in glomerular hyperfiltration and hypertrophy [17]. This can lead to secondary focal segmental glomerulosclerosis due to barotrauma in overworked nephrons [17]. RAAS activation, triggered by nephron loss and hypoperfusion, further aggravates injury [31]. Angiotensin II promotes vasoconstriction and fibrotic signaling (via transforming growth factor beta (TGF-β) and NF-κB), while aldosterone enhances inflammation and fibrosis [32]. Tubular cells, under sustained stress and hypoxia, may undergo EMT, contributing to fibrogenesis [32]. Additional contributors include aging, atherosclerosis, and genetic susceptibility. Notably, APOL1 risk variants in individuals of African ancestry heighten vulnerability to glomerulosclerosis and proteinuric kidney disease [33]. In malignant hypertension, severe vascular injury (fibrinoid necrosis, onion-skin lesions) can cause acute cortical infarction and rapid decline in renal function [34].

Pathophysiology of diabetic nephropathy

Diabetic nephropathy (DN), or diabetic kidney disease, develops from chronic hyperglycemia that induces both metabolic and hemodynamic disturbances [35]. Prolonged high glucose levels activate multiple harmful pathways [36]. One major mechanism is the formation of advanced glycation end-products (AGEs), which accumulate in the glomerular basement membrane (GBM) and mesangial matrix, increasing stiffness and triggering inflammation and fibrosis via receptors for advanced glycation endproducts (RAGE) [37]. Other metabolic pathways, such as the polyol and hexosamine pathways, and protein kinase C activation, also contribute to oxidative stress and pro-fibrotic signaling [38]. Hyperglycemia-driven oxidative stress damages endothelial cells, podocytes, and tubules, while also promoting chronic inflammation [36]. This amplifies fibrogenic signals, particularly through the upregulation of TGF-β and connective tissue growth factor (CTGF), driving mesangial expansion and extracellular matrix accumulation [39]. Hemodynamically, diabetes induces glomerular hyperfiltration, characterized by afferent arteriolar vasodilation and efferent vasoconstriction due to local nitric oxide effects and RAAS activation [40]. The resulting intraglomerular hypertension stretches capillary walls, disrupts the filtration barrier, and initiates protein leakage, podocyte stress, and eventual detachment [41]. Podocytes are especially vulnerable, hyperglycemia and AGEs reduce the expression of key slit diaphragm proteins, induce cytoskeletal disorganization, and lead to apoptosis or detachment [42]. Similarly, endothelial dysfunction impairs nitric oxide production and promotes vasomotor and coagulation imbalance, weakening the capillary barrier [43]. Over time, these injuries culminate in glomerulosclerosis and tubulointerstitial fibrosis [44]. Proteinuria itself accelerates damage by activating inflammatory responses in tubular cells [44]. This creates a self-perpetuating cycle where structural injury and functional decline reinforce one another [45]. Clinically, DN often follows a course beginning with hyperfiltration, progressing through microalbuminuria to overt proteinuria and reduced GFR [46]. Type 1 diabetes typically shows this progression after five to 10 years, while type 2 diabetes can vary widely due to undiagnosed disease [47]. Notably, some patients may have atypical presentations, such as non-proteinuric DN [48]. Genetic predispositions, though less clearly defined than in HN, may influence DN susceptibility, involving polymorphisms in RAAS and inflammatory pathway genes [49]. Ultimately, DN reflects the convergence of metabolic and hemodynamic stressors that drive renal injury, fibrosis, and progressive dysfunction [50]. Recent studies highlight the disruption of podocyte-endothelial crosstalk as a critical driver of DN. Hyperglycemia alters vascular endothelial growth factor (VEGF) signaling, leading to aberrant angiogenesis and increased glomerular permeability [51]. Podocyte apoptosis, triggered by oxidative stress and advanced glycation end products, results in loss of slit diaphragm integrity and progressive proteinuria [52]. Epigenetic modifications, including hypermethylation of anti-fibrotic genes and histone acetylation changes, have been implicated in sustaining inflammatory and fibrotic signaling even after glycemic control is achieved [53]. MicroRNAs such as miR-21 and miR-192 modulate extracellular matrix production and are emerging as potential biomarkers and therapeutic targets [54].

Histopathological features of diabetic nephropathy

DN presents distinct and well-characterized histological lesions, particularly in advanced disease [55]. The most consistent glomerular change is diffuse mesangial expansion, reflecting matrix accumulation with or without mesangial cell proliferation [56]. This occurs early in DN and correlates with proteinuria and declining glomerular filtration rate (GFR) [56]. In more advanced cases, nodular glomerulosclerosis (Kimmelstiel-Wilson nodules) may develop, which are rounded, acellular mesangial nodules located peripherally in the glomerular tuft, typically indicating severe and long-standing disease [57]. These nodules often form a background of diffuse expansion and are frequently associated with mesangiolysis and capillary microaneurysms [58]. As these lesions progress, they contribute to glomerular capillary obliteration and global sclerosis [58]. Another early hallmark is GBM thickening, first detectable by electron microscopy, becoming visible by light microscopy as disease progresses [59]. When seen alongside mesangial expansion, GBM thickening strongly supports DN [59]. Additional glomerular changes include hyaline deposits, such as fibrin caps and capsular drops, representing insudative protein leakage, common but not specific to DN [57]. Vascular involvement is striking in DN, with hyaline arteriolosclerosis affecting both afferent and efferent arterioles, a virtually diagnostic finding [60]. This bilateral involvement distinguishes DN from HN, where efferent hyalinosis is typically absent [61]. Tubulointerstitial changes evolve as disease advances [61]. While early DN may show minimal tubular injury, proteinuria and glomerulosclerosis eventually drive tubular atrophy, interstitial fibrosis, and peritubular capillary ischemia [62]. Inflammation may be mild initially but becomes more pronounced with disease progression [63]. The severity of tubulointerstitial damage often correlates more closely with renal function decline than glomerular pathology alone [63]. On ultrastructural examination, foot process effacement and expansion of the mesangial matrix are common [64]. Immunofluorescence is typically negative for immune complexes, helping to distinguish DN from primary glomerulonephritides, although nonspecific trapping can occur [65]. It is worth noting that nodular sclerosis is not exclusive to DN and can be mimicked by other conditions like light-chain deposition disease, but in diabetic patients, Kimmelstiel-Wilson nodules are considered diagnostic [66]. Clinically, DN typically presents with progressively worsening proteinuria, often reaching nephrotic range and steady GFR decline [67]. Diabetic retinopathy is often concurrent in type 1 diabetes and supportive of DN, although its absence (especially in type 2 diabetes) raises suspicion for alternative or superimposed kidney diseases [68]. Histologic classification systems, such as the Tervaert classification, stratify DN into four glomerular classes and score interstitial and vascular lesions [61]. In conclusion, key pathological features of DN include mesangial expansion, GBM thickening, nodular sclerosis, and arteriolosclerosis of both afferent and efferent vessels, often with accompanying tubulointerstitial fibrosis. These changes are strongly suggestive of diabetic etiology, particularly when nodules and efferent hyalinosis are present, distinguishing DN from other causes like HN.

Overlap between HN and DN

HN and DN share overlapping pathological features because both conditions injure the renal microvasculature and glomeruli [69]. It is common for patients, especially those with type 2 diabetes, to have both conditions simultaneously, making it difficult to determine which process predominates in kidney damage [3]. Both HN and DN produce glomerular sclerosis, arteriolar hyalinosis, and tubulointerstitial fibrosis [70]. In early or mild disease, biopsy findings can be nearly indistinguishable [70]. For instance, a patient with short-duration diabetes and hypertension might show mild diffuse mesangial expansion, vascular hyaline changes, and moderate interstitial fibrosis, lesions that resemble both early DN and HN [57]. Both diseases narrow afferent arterioles, leading to ischemic glomerular shrinkage and global sclerosis [71]. Hyperfiltration injury may occur in remaining nephrons, resulting in secondary focal segmental sclerosis (FSGS) [72]. Kidneys affected by both processes may exhibit mixed lesions, such as diabetic nodules in some glomeruli and ischemic collapse in others [73]. Certain pathological features help distinguish these entities [73]. Nodular mesangial sclerosis (Kimmelstiel-Wilson nodules) and efferent arteriole hyalinosis are characteristic of DN and rarely seen in pure HN [74]. Conversely, a predominance of ischemic global glomerulosclerosis with minimal mesangial expansion in a diabetic patient suggests hypertensive or arteriosclerotic damage as the primary etiology [75]. While mild mesangial expansion can occur in both conditions, extensive expansion with nodules strongly indicates DN [76]. Long-standing type 2 diabetes almost always coexists with hypertension, and the two factors accelerate renal injury [77]. Poor glycemic control exacerbates mesangial lesions, whereas uncontrolled blood pressure adds vascular and ischemic damage [78]. Both conditions present with bland urinary sediment and proteinuria, but DN typically produces heavier proteinuria, often reaching nephrotic range, while HN usually causes only low-grade proteinuria [79]. Imaging often reveals small, shrunken kidneys in both cases [80]. Mixed pathology is frequent [7]. Many biopsies from type 2 diabetics show overlapping diabetic and hypertensive lesions [81]. Moderate proteinuria with absent diabetic retinopathy may indicate a major hypertensive component, whereas advanced retinopathy strongly supports DN [82]. In such ambiguous cases, renal biopsy is essential to define the dominant process and guide treatment [82]. Table 1 illustrates the difference between HN and DN [2,46,57,71,83-93].

**Table 1:** Hypertensive Nephrosclerosis versus Diabetic Nephropathy GBM: Glomerular basement membrane; ESRD: end-stage renal disease

Aspect	Hypertensive Nephrosclerosis (HN)	Diabetic Nephropathy (DN)
Typical context	Long-standing hypertension; older patients; often with vascular comorbidities [2]	Long-standing diabetes (usually >5–10 years); often with coexisting hypertension (esp. in type 2 diabetes) [83]
Glomerular lesions	Global glomerulosclerosis from ischemia; segmental sclerosis in remnant nephrons; no nodular lesions [84]	Diffuse mesangial expansion; nodular Kimmelstiel-Wilson lesions in advanced cases; GBM thickening; hypertrophy [57]
Arteriolar changes	Hyaline arteriolosclerosis of afferent arterioles; efferent typically spared [71]	Hyaline arteriolosclerosis of both afferent and efferent arterioles; efferent involvement is classic [85]
Tubulointerstitial	Prominent fibrosis and tubular atrophy, often out of proportion to proteinuria [86]	Fibrosis and atrophy occur later, paralleling proteinuria and glomerular damage; early tubules may appear intact [46]
Proteinuria	Usually mild (<1-2 g/day); nephrotic range is rare unless coexisting disease present [87]	Often heavy; progresses from microalbuminuria to nephrotic-range proteinuria, especially with nodular sclerosis [88]
Hematuria	Minimal or absent; gross hematuria uncommon, except in malignant hypertension [86]	Minimal or absent; significant hematuria suggests another diagnosis [89]
Retinopathy correlation	May have hypertensive retinopathy; not specific [86]	Diabetic retinopathy often coexists and supports diagnosis; absence raises suspicion for non-diabetic kidney disease [90]
Rate of progression	Typically slow with blood pressure control; faster with severe or malignant hypertension [91]	Progressive decline unless hyperglycemia and hypertension are well controlled; risk of ESRD is high [92]
Prognosis	Favorable with control; higher ESRD risk in patients with APOL1 risk alleles [91]	High risk of ESRD and cardiovascular events; improved with early, intensive interventions [93]

**Figure 1:**
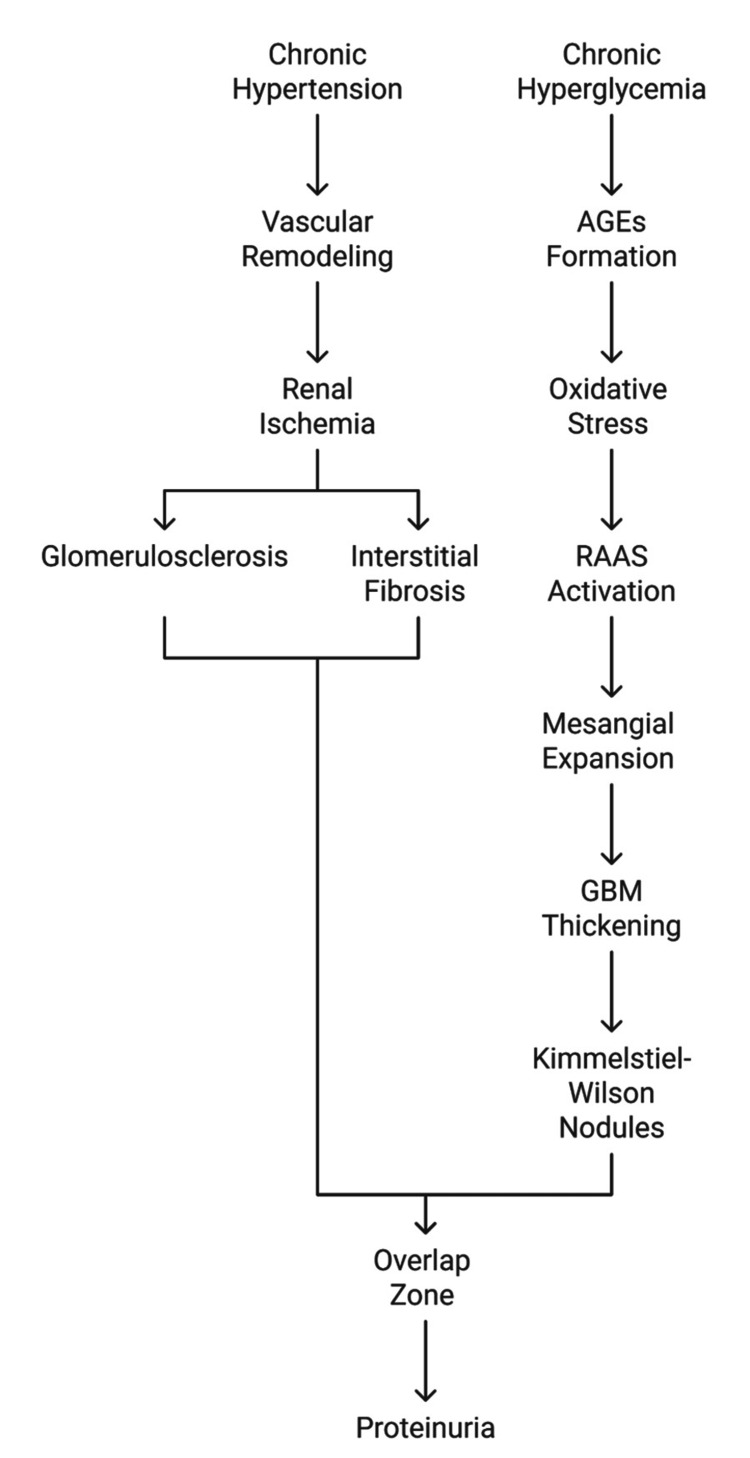
Pathophysiological pathways of hypertensive nephrosclerosis (HN) and diabetic nephropathy (DN), highlighting points of convergence. AGEs: advanced glycation end-products; RAAS: Renin-angiotensin-aldosterone system; GBM: glomerular basement membrane. Flowchart credit: Dr. Karis Khattab.

Although HN and DN have distinct initiating mechanisms, both ultimately converge on similar pathways of chronic kidney injury, including glomerulosclerosis, interstitial fibrosis, and proteinuria (Figure 1).

Quantitative morphometric analyses from biopsy studies reveal that in type 2 diabetics with coexistent hypertension, approximately 40%-60% of glomeruli may display ischemic global sclerosis consistent with HN, while the remainder exhibit mesangial expansion or nodular lesions characteristic of DN [61]. These proportions vary with disease duration and glycemic control, underscoring the need for integrated clinico-pathologic assessment. Clinical prediction models incorporating the duration of diabetes, degree of proteinuria, and presence of retinopathy have shown moderate accuracy in differentiating DN from non-diabetic kidney disease, but biopsy remains the gold standard [94].

Diagnostic challenges and approaches

Distinguishing HN from DN is a frequent clinical dilemma, especially when both conditions coexist [95]. Since both hypertension and diabetes commonly overlap and share pathological features, clinicians must rely on clinical context, lab findings, and sometimes kidney biopsy to differentiate the causes of renal dysfunction [96]. DN generally develops after at least five years of diabetes, particularly in type 1 patients [97]. Thus, kidney disease presenting early in the course of diabetes, or even preceding the diagnosis, raises the possibility of HN or another non-diabetic kidney disease [3]. Diabetic microvascular complications such as retinopathy and neuropathy are suggestive of DN, while their absence, especially in type 1 diabetics with kidney disease, may indicate an alternative etiology [98]. Proteinuria severity also aids differentiation: nephrotic-range proteinuria is much more typical of DN, whereas HN usually causes only mild proteinuria [55]. A diabetic patient with declining GFR but low-level proteinuria is more likely to have HN [99]. Laboratory and imaging findings can help, though they often lack specificity [100]. Both conditions usually result in bland urinary sediment [99]. If active sediment is present, such as hematuria with dysmorphic red cells or casts, other diagnoses should be considered [101]. Biomarkers and imaging advances are under investigation but have yet to gain routine clinical application [102]. Ultrasound findings are generally non-specific, as both conditions in late stages can result in small, echogenic kidneys [103]. Renal biopsy remains the gold standard when clinical features are ambiguous [104]. Classic histologic features of DN include nodular glomerulosclerosis and efferent arteriolar hyalinosis, whereas HN is characterized by global glomerulosclerosis, arteriosclerosis, and ischemic changes without nodules [57]. Biopsy is particularly valuable in atypical cases, such as nephrotic-range proteinuria without retinopathy, sudden decline in function, or presence of hematuria [105]. In many diabetic patients with proteinuria, biopsy has revealed non-diabetic renal disease (NDRD) or mixed pathology, which can alter management [106]. That said, biopsies are not without risk, and not every diabetic patient with CKD requires one [107]. If the clinical course aligns with classic DN, that is, long-standing diabetes, proteinuria, presence of retinopathy, a presumptive diagnosis may be sufficient [108]. However, when atypical features emerge, biopsy should be considered [109]. In hypertensive patients without diabetes, HN is often diagnosed by exclusion, especially in those with mild proteinuria [8]. This can lead to misclassification, as conditions like primary FSGS or glomerulonephritis may be the actual cause [8]. In African American patients, APOL1-associated nephropathy may be misdiagnosed as HN when it is a primary glomerular disease [110]. Genetic testing for APOL1 risk alleles may aid in clarifying the diagnosis in such cases [111]. Ongoing research into noninvasive diagnostic tools offers future promise [112]. Urinary proteomics and advanced imaging methods (e.g., MRI for perfusion or stiffness) may eventually help distinguish DN from HN [113]. Clinical scoring systems using variables like retinopathy, proteinuria, and diabetes duration may also guide biopsy decisions [114]. While no single test is definitive outside of biopsy, combining clinical clues improves diagnostic confidence [114].

Prognostic and therapeutic implications

Accurately distinguishing whether CKD is due to HN, DN, or both has important prognostic and therapeutic implications [61]. DN generally progresses faster to ESRD than HN, especially with poor glycemic control [115]. Overt DN with significant proteinuria and nodular glomerulosclerosis may reach ESRD within a few years, whereas isolated HN often declines more slowly with adequate blood pressure control [116]. Both DN and HN require strict blood pressure control [117]. In DN, the target BP is <130/80 mmHg, with angiotensin-converting enzyme (ACE) inhibitors or angiotensin II receptor blockers (ARBs) preferred for their renoprotective effects (Reduction of Endpoints in Non-Insulin-Dependent Diabetes Mellitus with the Angiotensin II Antagonist Losartan (RENAAL) and Irbesartan Diabetic Nephropathy Trial (IDNT) trials) [118]. HN also benefits from ACE inhibitors (African American Study of Kidney Disease and Hypertension (AASK) trial), especially in proteinuric cases, though other antihypertensives are suitable for non-proteinuric HN [119]. Glycemic control is central to DN (Diabetes Control and Complications Trial (DCCT) and United Kingdom Prospective Diabetes Study (UKPDS)), with an HbA1c goal around 7%, while metabolic risk factors should be addressed in HN to lower cardiovascular risk [120]. Recent advances for DN include sodium-glucose transport protein 2 (SGLT2) inhibitors (empagliflozin, dapagliflozin; Canagliflozin and Renal Events in Diabetes with Established Nephropathy Clinical Evaluation (CREDENCE), Dapagliflozin and Prevention of Adverse Outcomes in Chronic Kidney Disease (DAPA-CKD) trials), finerenone (Finerenone in Reducing Kidney Failure and Disease Progression in Diabetic Kidney Disease (FIDELIO-DKD) trial), and glucagon-like peptide-1 (GLP-1) receptor agonists, all improving renal and cardiovascular outcomes [121]. These are not indicated for isolated HN but highlight the value of accurate diagnosis. Lifestyle interventions are important in both: HN focuses on salt restriction and weight reduction, DN on glycemic targets and moderated protein intake; smoking cessation is universally advised [122]. Landmark randomized trials have quantified the renal benefits of targeted therapies in DN. In the RENAAL trial, losartan reduced the risk of doubling of serum creatinine, ESRD, or death compared with placebo [123]. The CREDENCE trial demonstrated a relative risk reduction in the composite renal endpoint with canagliflozin in patients with type 2 diabetes and albuminuric CKD [124]. Similarly, finerenone in the FIDELIO-DKD trial significantly reduced kidney failure or sustained estimated glomerular filtration rate (eGFR) decline [125]. For HN, the AASK trial showed that intensive blood pressure (BP) lowering with ramipril slowed GFR decline more effectively than beta-blockers or calcium channel blockers, particularly in proteinuric patients [126]. These data reinforce the need for accurate etiologic diagnosis to maximize therapeutic benefit. Misdiagnosis can delay key therapies or overlook reversible disease, making careful clinical evaluation, biopsy when needed, essential [127]. Many patients have overlapping pathology, benefiting from tight control of both blood pressure and glucose, RAAS inhibition, SGLT2 inhibitors when indicated, and statin therapy [128]. Treatment focus shifts depending on the dominant condition: DN requires intensive glycemic control and newer agents, while HN prioritizes vascular risk reduction and stringent BP goals [129]. Ultimately, distinguishing DN from HN enables tailored therapy, improving renal and cardiovascular outcomes.

Future directions

Ongoing research is improving the ability to distinguish and treat HN and DN, with emphasis on early detection and personalized therapy [130]. Promising developments include noninvasive biomarkers: proteomic studies show DN-specific urinary peptides (collagen fragments, nephrin, podocin), while tubular injury markers like kidney injury molecule-1 (KIM-1) may indicate ischemic injury seen in HN [131]. Although not yet in routine use, combined biomarkers may reduce the need for biopsy [132]. Advanced imaging, such as MRI for perfusion, fibrosis, and microvascular density, may differentiate DN’s diffuse microangiopathy from HN’s patchy ischemia; AI-enhanced retinal imaging may serve as a surrogate for systemic microvascular injury [133]. Digital pathology and artificial intelligence are enabling automated recognition of DN and HN histologic patterns and quantification of lesions (e.g., nodular sclerosis vs ischemic scarring), potentially refining prognosis and therapy [134]. Genomics also offers insights, APOL1 risk variants clarify HN etiology in African Americans, while polygenic risk scores may predict DN susceptibility [135]. Therapies are becoming more targeted: in DN, SGLT2 inhibitors, finerenone, and agents against glycation, inflammation, and fibrosis are reshaping management; in HN, anti-inflammatory and microvascular function, enhancing drugs (nitric oxide donors, endothelin antagonists) are under study [136,137]. Classification systems may evolve, with “hypertensive nephrosclerosis” replaced by more precise terms like “APOL1-associated nephropathy” or “arterionephrosclerosis of aging” [138]. Efforts in early detection continue: microalbuminuria screening in diabetes allows earlier ACE inhibitor use, and emerging biomarkers in hypertension may soon identify subclinical kidney damage for timely intervention [139]. Multi-omics approaches integrating genomics, transcriptomics, and proteomics hold promise for distinguishing DN from HN in clinically ambiguous cases [140]. Early studies suggest that combining urinary proteomic signatures with genetic risk scores, such as APOL1 or polygenic DN scores, may improve diagnostic accuracy without biopsy [140]. Artificial intelligence applied to digital pathology has achieved >90% accuracy in differentiating nodular sclerosis from ischemic glomerulosclerosis in pilot studies [141]. At the public health level, reducing salt and sugar intake within the population, coupled with early screening for albuminuria and hypertension, remains a cost-effective prevention strategy [142].

## Conclusions

Hypertensive nephrosclerosis and diabetic nephropathy are the leading, and often coexisting, contributors to chronic kidney disease, sharing converging mechanisms of injury such as glomerulosclerosis, arteriolar hyalinosis, and interstitial fibrosis. Despite differing etiologies - hypertension versus hyperglycemia - differentiating between them is essential, as it directly influences prognosis and therapeutic decisions. Classic histologic features such as Kimmelstiel-Wilson nodules and efferent arteriolar hyalinosis, along with clinical indicators like long-standing diabetes, retinopathy, and heavy proteinuria, support a diagnosis of diabetic nephropathy. Conversely, ischemic glomerular scarring with mild proteinuria in a hypertensive patient may favor hypertensive nephrosclerosis. In many type 2 diabetics, overlapping pathology necessitates a dual-focused management approach targeting both glycemic and blood pressure control. When diagnostic ambiguity persists, renal biopsy remains the gold standard. Therapy should be individualized: RAAS blockade and optimal blood pressure management for all, augmented by glycemic control and disease-modifying agents such as SGLT2 inhibitors or finerenone in confirmed diabetic kidney disease. An accurate identification of the underlying pathology is key to guiding appropriate intervention and improving outcomes. Future innovations in biomarkers, imaging, and genomics may eventually allow for noninvasive differentiation and earlier risk stratification. Until then, aggressive control of cardiovascular and metabolic risk factors remains the cornerstone of prevention and management. A refined understanding of the overlapping but distinct pathology in HN and DN will enhance personalized care and reduce the burden of CKD.
